# Regulation of Alr1 Mg Transporter Activity by Intracellular Magnesium

**DOI:** 10.1371/journal.pone.0020896

**Published:** 2011-06-28

**Authors:** Phaik Har Lim, Nilambari P. Pisat, Nidhi Gadhia, Abhinav Pandey, Frank X. Donovan, Lauren Stein, David E. Salt, David J. Eide, Colin W. MacDiarmid

**Affiliations:** 1 Department of Molecular and Human Genetics, Baylor College of Medicine, Houston, Texas, United States of America; 2 Department of Genetics, Washington University School of Medicine, St. Louis, Missouri, United States of America; 3 Department of Pharmacology and Pharmacokinetics, Regeneron Pharmaceuticals, Inc., Tarrytown, New York, United States of America; 4 Bijvoet Center for Biomolecular Research, Faculty of Science, Utrecht University, Utrecht, The Netherlands; 5 Cancer Genetics Branch, National Human Genome Research Institute, National Institutes of Health, Bethesda, Maryland, United States of America; 6 Department of Pharmacology and Toxicology, Medical College of Wisconsin, Wauwatosa, Wisconsin, United States of America; 7 School of Biological Sciences, University of Aberdeen, Aberdeen, Scotland, United Kingdom; 8 Department of Nutritional Sciences, University of Wisconsin-Madison, Madison, Wisconsin, United States of America; Auburn University, United States of America

## Abstract

Mg homeostasis is critical to eukaryotic cells, but the contribution of Mg transporter activity to homeostasis is not fully understood. In yeast, Mg uptake is primarily mediated by the Alr1 transporter, which also allows low affinity uptake of other divalent cations such as Ni^2+^, Mn^2+^, Zn^2+^ and Co^2+^. Using Ni^2+^ uptake to assay Alr1 activity, we observed approximately nine-fold more activity under Mg-deficient conditions. The *mnr2* mutation, which is thought to block release of vacuolar Mg stores, was associated with increased Alr1 activity, suggesting Alr1 was regulated by intracellular Mg supply. Consistent with a previous report of the regulation of Alr1 expression by Mg supply, Mg deficiency and the *mnr2* mutation both increased the accumulation of a carboxy-terminal epitope-tagged version of the Alr1 protein (Alr1-HA). However, Mg supply had little effect on *ALR1* promoter activity or mRNA levels. In addition, while Mg deficiency caused a seven-fold increase in Alr1-HA accumulation, the N-terminally tagged and untagged Alr1 proteins increased less than two-fold. These observations argue that the Mg-dependent accumulation of the C-terminal epitope-tagged protein was primarily an artifact of its modification. Plasma membrane localization of YFP-tagged Alr1 was also unaffected by Mg supply, indicating that a change in Alr1 location did not explain the increased activity we observed. We conclude that variation in Alr1 protein accumulation or location does not make a substantial contribution to its regulation by Mg supply, suggesting Alr1 activity is directly regulated via as yet unknown mechanisms.

## Introduction

Magnesium (Mg) is the fourth most abundant cation in the body, and the second most abundant within cells (after potassium) [1]. Mg is a critical co-factor for hundreds of enzymes [2], [3], and utilized by twice as many metalloenzymes as zinc [4]. In environments with abundant Mg, its tendency to over-accumulate in cells can challenge homeostatic mechanisms [5]. Conversely, limited Mg supply can also constrain growth. In bacteria, adaptation to Mg deficiency is essential for pathogenicity and survival within macrophages [6]. In humans, gut or renal disorders can affect Mg homeostasis by altering rates of absorption or excretion, as can drugs such as diuretics [7], [8]. Low dietary Mg intake has been associated with cardiovascular disease, as well as the development of type II diabetes [9], [10], hypertension [11], and stroke [12]. Cytosolic Mg is distributed between a large pool bound to proteins, nucleic acids and small molecules, and a smaller, regulated pool of free-ionized Mg [13], [14]. Regulation of the cytosolic free-ionized Mg concentration is likely achieved by three major mechanisms: control of uptake systems, efflux from the cell, and sequestration within organelles. Despite the importance of this cation however, we are only beginning to understand the molecular basis of Mg homeostasis in eukaryotic cells.

The CorA (or Metal Ion Transporter) superfamily is an important group of Mg transporters in prokaryotes and eukaryotes [15], [16]. Eukaryotic CorA proteins have diversified in function, facilitating both Mg uptake and distribution between subcellular compartments. One subfamily includes the yeast Mrs2 protein, which supplies Mg to the mitochondrial matrix [17]. Vertebrate genomes include mitochondrial proteins of similar function to yeast Mrs2 [18], [19], while higher plant Mrs2 homologs have diverged to function in additional cellular compartments [20], [21], [22], [23]. A second major branch of the eukaryotic CorA proteins is defined by the yeast plasma membrane Alr1 and Alr2 proteins [24], [25]. Loss of function mutations in Alr1 reduced Mg uptake and induced a growth defect that was suppressible by excess Mg [24], [26]. Alr2 makes a minor contribution to Mg homeostasis, due to low expression and activity [25], [27]. The Alr1 branch of the CorA proteins includes a subgroup defined by Mnr2 [28], a vacuolar membrane protein required for access to intracellular Mg stores [29]. Mutants lacking Mnr2 displayed a growth defect and accumulated a higher intracellular Mg content in Mg-deficient conditions. As Alr1 and Mnr2 both supply Mg to the cytosol, the regulation of these proteins is likely to be of central importance to cytosolic Mg homeostasis.

The expression of many microbial metal cation transporters is regulated by availability of their substrates [30]. This regulation serves to increase the supply of essential cations under deficient conditions, while preventing the potentially deleterious effects of overaccumulation. Regulation of Mg-transporter expression has also been shown to contribute to microbial Mg homeostasis. The bacterial MgtA and MgtB high affinity Mg uptake systems are regulated by external and cytosolic Mg supply, both transcriptionally via the activity of the two-component Mg sensor PhoP/Q, and translationally via direct binding of Mg to the MgtA mRNA leader sequence [31], [32]. In contrast to these regulated systems, gene expression of microbial CorA proteins is generally independent of Mg supply [33]. The yeast *ALR1* and *ALR2* genes are apparent exceptions to this rule, as their expression was reported to vary with Mg supply [24], [25], although the mechanism of this regulation was not determined.

Post-translational regulation of transporter stability in response to substrate level is also a common feature of microbial metal homeostasis [34], [35], [36]. For example, the high-affinity zinc transporter Zrt1 accumulates to a high level in zinc-deficient yeast cells. Upon exposure of cells to zinc-replete conditions, Zrt1 is rapidly internalized and routed to the vacuole for degradation. This process was dependent on the End3, Rsp5 and Pep4 proteins, which encode factors required for Zrt1 endocytosis, ubiquitination and vacuolar degradation respectively [35], [37]. The Alr1 protein was also reported to be post-translationally regulated in response to Mg supply [24]. An epitope-tagged version (Alr1-HA) was rapidly degraded when Mg-deficient cells were shifted to Mg-replete conditions, and this process was also dependent on End3, Rsp5 and Pep4, suggesting that Alr1 stability was regulated by ubiquitin-dependent endocytosis and degradation. In addition to being required for the regulation of some transporters, Pep4 and Rsp5 also enable the degradation of some aberrantly folded plasma membrane proteins (*e.g.* Pma1-7 and Ste2-3) [38], [39], [40]. Ubiquitination of these proteins has been shown to occur early in the secretory pathway, resulting in their direct trafficking to the vacuole compartment without transit through the plasma membrane.

We previously reported that the *mnr2* mutation reduced tolerance to several divalent cations, while at the same time increasing their accumulation by yeast cells [29]. The latter effect was exacerbated by growth in Mg-deficient conditions. We suspected that these phenotypes were due to an increase in the expression of a non-specific divalent cation transporter in the *mnr2* mutant, for which the Alr proteins were candidates [26]. The observation that *ALR1* and *ALR2* gene expression is Mg-regulated [24], [25] suggested that yeast may respond to a perceived Mg-deficiency associated with the *mnr2* mutation by inducing *ALR* gene expression. In this study, we tested this hypothesis by determining the effect of the *mnr2* mutation on Alr1 activity and the accumulation of the Alr1 protein. We provide the first direct evidence that the activity of the Alr1 system is Mg-responsive. In addition, we report elevated Alr1 activity in an *mnr2* mutant, consistent with perturbation of Mg homeostasis. However, a previous report of the Mg-regulated expression of the *ALR1* gene and Alr1 protein stability [24] was not supported by our experiments, suggesting that Alr1 activity is regulated by some other mechanism. We also propose a model to explain the aberrant behavior of epitope-tagged Alr1-HA.

## Results

### Measurement of Alr activity

**Figure 1:**
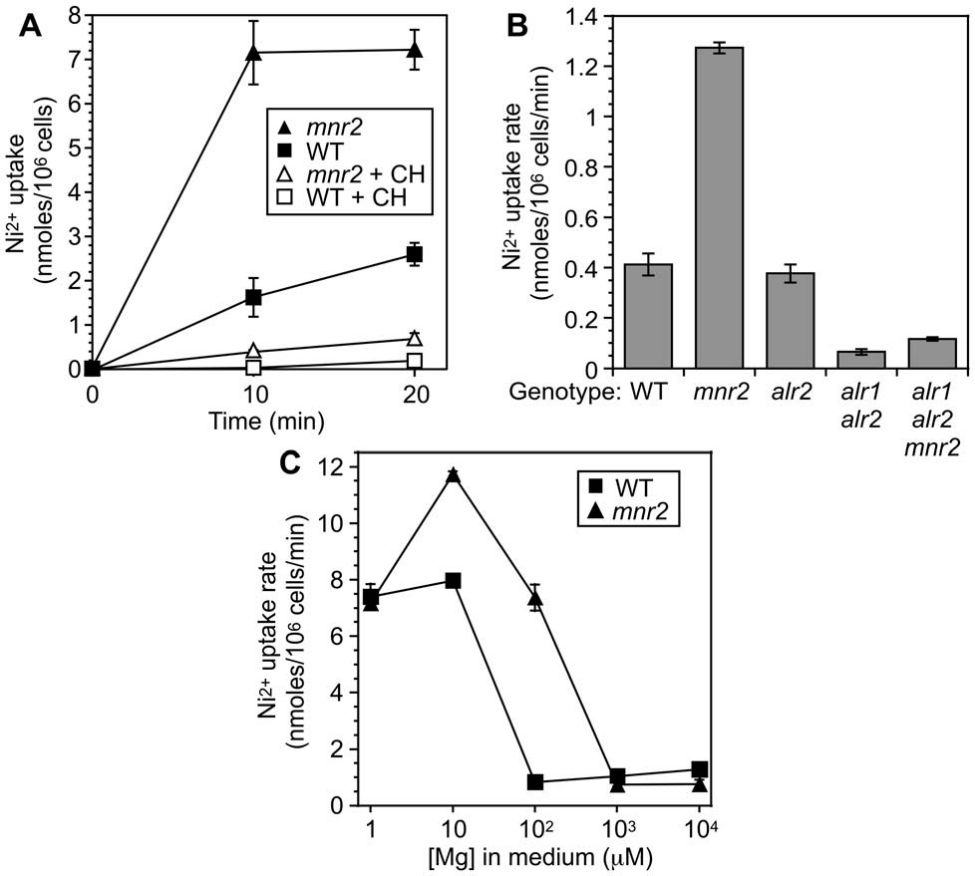
Effect of Mg supply and the *mnr2* mutation on Alr system activity. (**A**) Effect of cobalt(III)hexaammine chloride (CH) on Ni^2+^ uptake activity. WT (DY1457) and *mnr2* (NP4) strains were grown to log phase in YPD medium. At zero time, cells were added to uptake buffer containing 100 µM NiCl_2_, with or without 100 µM CH inhibitor. At the indicated times, aliquots were removed and processed for measurement of nickel content by ICP-MS, as described in Materials and Methods. (**B**) Rate of CH-inhibited Ni^2+^ uptake by WT (DY1457), *mnr2* (NP4), *alr2* (NP27), *alr1 alr2* (NP14) and *alr2 alr2 mnr2* (NP20) strains. Assays were performed as described in (A), except strains were grown to log phase in YPD+50 mM MgCl_2_. Nickel content was determined zero and 5 min after addition of 100 µM NiCl_2_, and initial Ni^2+^ content subtracted from 5 min values. (**C**) Regulation of the Alr systems by Mg supply. DY1457 (WT) and *mnr2* (NP4) strains were grown to log phase in SC medium, washed to remove excess Mg, and incubated for 6 h in LMM with the indicated Mg concentration. Cells were washed, resuspended in uptake buffer, and incubated with 100 µM Ni^2+^ for 1 min. Initial Ni^2+^ content was subtracted from 1 min values to give the results shown. For all graphs, values are means of four replicates, and error bars indicate +/−1 SEM.

To determine the effect of Mg supply and the *mnr2* mutation on the regulation of the Alr systems, we initially attempted to measure the rate of Mg uptake by cells grown over a range of Mg concentrations, using atomic absorption spectroscopy to measure the change in Mg content of cells when subsequently supplied with a dose of excess Mg (AAS) [24], [41], [42]. However, it was not possible to accurately measure uptake by wild-type cells grown in relatively Mg-replete conditions, as the amount of Mg accumulated was much less than the initial content (data not shown) [43]. Comparison of wild-type and *mnr2* strains was also not possible with this method, as *mnr2* cells have a high Mg content in both replete and deficient conditions [29]. As an alternative, we investigated the use of surrogate divalent cations (Co^2+^, Mn^2+^, Ni^2+^ and Zn^2+^) to measure Alr system activity, using Inductively Coupled Plasma-Mass Spectrometry (ICP-MS) to monitor the uptake of these cations. Of these metals, we selected Ni^2+^ as the most suitable tracer. This decision was in part based on the observation that inhibitors of the Alr proteins and other CorA transporters, such as aluminum ion [23], [44], cobalt(III)hexaammine chloride (CH) [23], [45], [46], and Mg ion [23], [43], all strongly inhibited Ni^2+^ uptake (**Figure 1** and data not shown), suggesting Ni^2+^ uptake was primarily mediated by the Alr systems. Strong additional support for Alr-mediated Ni^2+^ uptake comes from the observation that Ni^2+^ competitively inhibited Mg uptake by *Saccharomyces cerevisiae* [43], and that a mutation inactivating the *Schizosaccharomyces pombe ALR1* homolog also reduced Ni^2+^ uptake [47].

To determine the basic parameters of Ni^2+^ uptake by yeast, we supplied strains with 100 µM Ni^2+^ in the presence or absence of 100 µM CH (**Figure 1A**) and followed accumulation over a 20 min time course. Substantial Ni^2+^ uptake by a wild-type strain was observed after 10 minutes of incubation, and the rate slowed only slightly after 20 min. Addition of CH at the start of the time course efficiently suppressed this uptake, consistent with Ni^2+^ influx via the Alr systems. We then examined the effect of inactivating plasma membrane Mg uptake systems on Ni^2+^ uptake. If the Alr systems mediate the majority of Ni^2+^ uptake, the rate of uptake should be substantially reduced by the inactivation of these transporters. To test this prediction, we determined the rate of Ni^2+^ uptake by a set of Mg transporter mutants (**Figure 1B**). Since *alr1* mutants exhibit a growth defect under normal conditions, all strains were cultured in YPD medium supplemented with excess Mg (50 mM). The Ni^2+^ uptake rate was reduced by 85% in an *alr1 alr2* strain, indicating that the bulk of Ni^2+^ uptake activity was contributed by these systems. Inactivating the *ALR2* gene alone had a negligible effect on Ni^2+^ uptake, indicating that the majority of this activity was dependent on *ALR1*. Addition of equimolar CH inhibited 94% of Ni uptake by wild-type cells, but was less inhibitory to *alr1 alr2* mutants, reducing Ni uptake by 81% for the *alr1 alr2* strain and 76% for the triple mutant (data not shown). These observation suggest that CH is not completely specific to the Alr systems, but may also partially inhibit residual Ni uptake systems revealed by inactivation of the Alr systems. However, the genetic data indicate that these systems make little contribution to total Ni uptake by yeast.

### Effect of Mg supply on Alr1 activity

The previously reported effect of Mg supply on *ALR1* expression [24] suggested that Mg-deficient yeast would display increased Ni^2+^ uptake activity. To test this prediction, wild-type cells were grown to log phase in standard SC medium, then transferred to low magnesium medium (LMM) supplemented with a range of Mg concentrations (1 µM–10 mM Mg) and incubated for a further 6 h prior to assaying Ni^2+^ uptake. In wild-type yeast, activity was relatively low in cells grown in 100 µM–10 mM Mg, but increased substantially as the Mg supply decreased to 10 µM or below (**Figure 1C**). Together, these observations strongly suggested that yeast cells responded to Mg deficiency by increasing Alr1 activity.

We also examined the effect of the *mnr2* mutation on Alr1 activity. The *mnr2* mutation blocks access to intracellular Mg stores required to maintain homeostasis under deficient conditions [29]. If Alr1 activity was regulated by cytosolic Mg concentration, we suspected that by reducing cytosolic Mg availability, the *mnr2* mutation might enhance Alr1 activity. Consistent with this expectation, Ni^2+^ uptake by *mnr2* cells grown in YPD was rapid and essentially complete after 10 minutes (**Figure 1A**). Addition of CH inhibitor reduced this activity by 96% (data not shown) suggesting that the increase was a consequence of Alr1 activation, rather than the activation of an independent Ni^2+^ transporter. As a further test of this model, we examined the effect of inactivating both Alr proteins on Ni^2+^ uptake by the *mnr2* mutant (**Figure 1B**). The *mnr2* mutation alone was again associated with increased uptake, but combining *alr1* and *alr2* mutations with the *mnr2* mutation eliminated the majority of this activity. Together, these findings indicate that the increased Ni^2+^ uptake associated with *mnr2* was not due to the activation of a novel Ni^2+^ transport system, but occurred as a consequence of a specific increase in Alr system activity. Since the bulk of this activity is contributed by Alr1, we will refer to this Ni^2+^ uptake activity as Alr1 activity in subsequent discussions.

Like the wild-type, the *mnr2* mutant displayed Mg-responsive Alr1 activity, with the highest activity seen in cells supplied with 10 µM Mg (**Figure 1C**). Alr1 activity was similar to wild-type in cells supplied with 1 mM Mg or higher, but mutant cells supplied with 10–100 µM Mg displayed a substantial increase in activity over the wild-type. In mutant cells supplied with 1 µM Mg however, activity was similar to wild-type. Although the reason for this decrease relative to the wild-type is unclear, it is possible that at this low concentration, the *mnr2* mutation enhanced the general negative effects of severe Mg deficiency on yeast physiology.

Taken together, the above observations suggest two major conclusions. First, yeast respond to reduced Mg supply by increasing Alr1 activity, presumably to capture more Mg from the environment. This effect occurred both in response to a decrease in external concentration, and to a loss of access to intracellular stores, suggesting that the major “pool” of Mg sensed by the yeast cell is cytosolic. Second, the *mnr2* mutation did not eliminate regulation by Mg supply, as *mnr2* cells supplied with 10 mM Mg still displayed less Alr1 activity than cells supplied with 10 µM Mg. For this reason, Mnr2 is unlikely to represent a component of a pathway required for Alr1 regulation. Instead, these results support a more general role for Mnr2 in the maintenance of Mg homeostasis [29].

### Mg-responsive accumulation of the Alr1-HA protein

**Figure 2:**
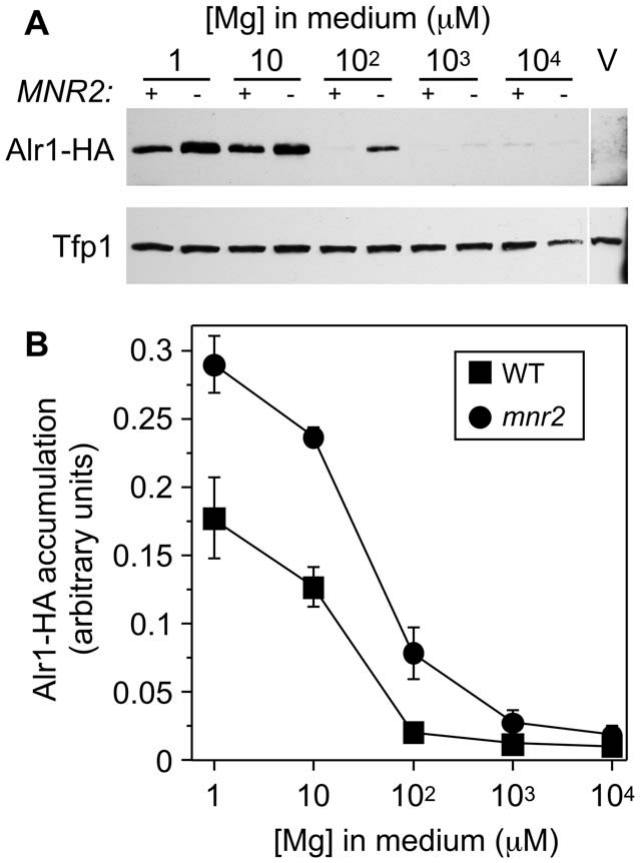
Alr1-HA accumulation varies with Mg supply and *MNR2* genotype. (**A**) Alr1-HA accumulation in WT (DY1457) and *mnr2* (NP4) strains transformed with YIpALR1-HA were grown to log phase in LMM-ura with the indicated concentration of Mg. Proteins were detected by immunoblotting with anti-HA and anti-Tfp1 antibodies. *MNR2* genotype is indicated (+ or −). DY1457 transformed with empty vector was included to show antibody specificity (pFL38, V). (**B**) Quantitation of Alr1-HA accumulation. Immunoblot band density was normalized to the total Alr1-HA signal detected in both strains for each replicate. Values are average of five independent experiments, including the result shown in A. Error bars show +/−1 SEM.

A previous report [24] indicated that the accumulation of a C-terminally epitope-tagged version of the Alr1 protein (Alr1-HA) was influenced by Mg supply. Since this regulation might explain the effect of Mg supply and the *mnr2* mutation on Alr1 activity, we performed experiments to verify the effect of Mg on Alr1-HA accumulation. Wild-type and *mnr2* cells expressing Alr1-HA were supplied with a range of Mg concentrations, from replete to deficient (<100 µM), and Alr1-HA detected by immunoblotting of total protein extracts (**Figure 2A**). In the wild-type strain, we observed a 19-fold increase in the Alr1-HA content of wild-type cells supplied with 1 µM vs 10 mM Mg (**Figure 2B**), confirming that Alr1-HA accumulation was responsive to Mg supply. In the *mnr2* mutant strain, Alr1-HA accumulated to a higher level than in the wild-type. This effect was seen at all Mg concentrations tested, but was most pronounced at intermediate concentrations (for example, Alr1-HA accumulation was approximately 4-fold higher in cells supplied with 100 µM Mg, **Figure 2B**). These observations confirmed a previous report that yeast cells respond to Mg deficiency by elevating the accumulation of the Alr1-HA protein. In addition, the effect of the *mnr2* mutation suggested that this process is responsive to the intracellular Mg concentration. Subsequent experiments were performed to examine the mechanism of this apparent regulation.

### Effect of Mg supply on ALR1 gene expression

**Figure 3:**
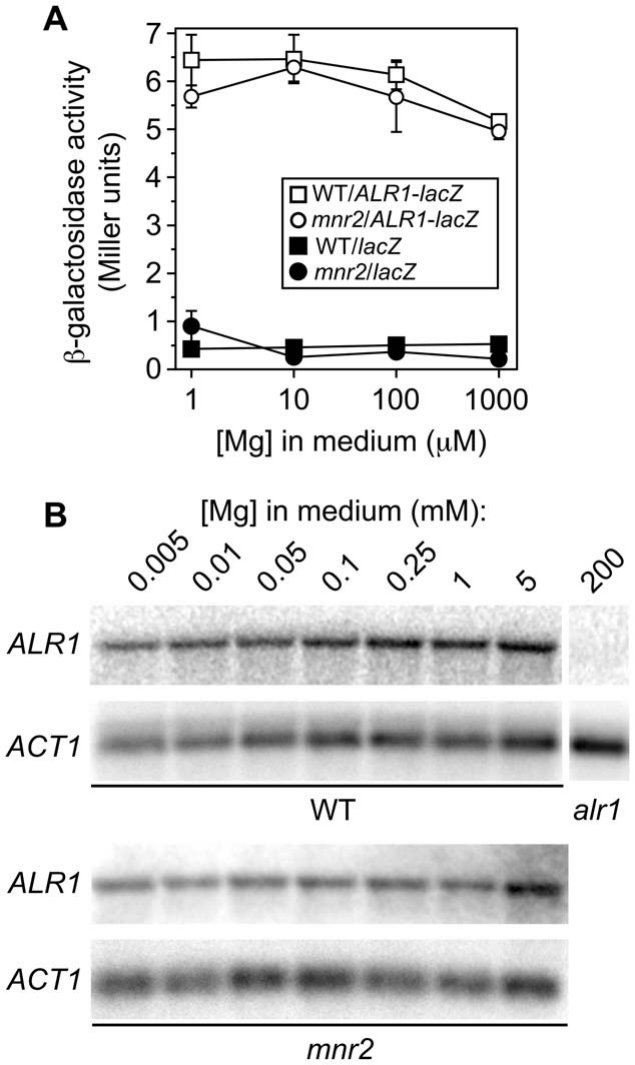
Effect of Mg supply and the *mnr2* mutation on *ALR1* gene expression. (**A**) WT (DY1457) and *mnr2* (NP4) strains transformed with YEpALR1-lacZ (*ALR1-lacZ*) or control promoterless *lacZ* vector (YEp353, *lacZ*) were grown to log phase in LMM with the indicated Mg concentration, and β-galactosidase activity determined. Error bars indicate +/−1 SEM (six replicates). (**B**) Effect of Mg supply and the *mnr2* mutation on *ALR1* mRNA accumulation. mRNA was purified from WT (DY1457) or *mnr2* mutant (NP4) cultures grown to log phase in LMM containing the indicated Mg concentration and subjected to Northern analysis. Blots were probed with a ^32^P-labeled PCR product of the *ALR1* gene, stripped, and reprobed with a ^32^P-labeled PCR product of the *ACT1* gene. mRNA from an *alr1*::*HIS3* mutant (NP10) grown in YPD+200 mM Mg was included as a control for *ALR1* probe specificity (*alr1*).

One potential explanation for the results shown in **Figure 1** and **Figure 2** is that yeast cells can respond to Mg deficiency by inducing *ALR1* gene expression, as previously reported [24]. To determine if the *ALR1* gene was transcriptionally regulated, we measured the activity of an *ALR1* promoter-*lacZ* fusion in wild-type and *mnr2* mutant strains (**Figure 3A**). The *mnr2* strain was included in order to determine if the higher Alr1 activity seen in this strain could be explained by higher *ALR1* gene expression. The *ALR1* promoter-*lacZ* fusion drove higher *lacZ* expression than the promoter-less *lacZ* construct (YEp353), indicating that the promoter was functional. However, reporter activity was only slightly increased by Mg-deficiency in both the wild-type and *mnr2* strains, and was slightly reduced by the *mnr2* mutation. Neither observation was consistent with substantial transcriptional regulation of *ALR1* gene expression by Mg supply.

Given the previous report of Mg-responsive *ALR1* transcript accumulation [24], the above observation suggested that the *ALR1* mRNA might be subject to Mg-regulated degradation. To test this model, we directly examined the effect of Mg supply on *ALR1* transcript abundance, via Northern analysis of *ALR1* mRNA (**Figure 3B**). In mRNA of the wild-type strain, a single band was detected by the *ALR1* probe (lanes 1–7, WT). This band was absent from mRNA of the control *alr1* strain (lane 8), indicating that the probe was specific for *ALR1*. Consistent with the *lacZ* reporter assays, no significant variation in *ALR1* transcript level was observed with changing Mg supply, indicating that *ALR1* gene expression was not affected by Mg availability. As expected, expression of two control transcripts (*ACT1*, **Figure 3B** and *RPL3*, data not shown) was also insensitive to Mg supply. We also examined *ALR1* transcript accumulation in an *mnr2* mutant under the same conditions, and observed no change in the effect of Mg on transcript accumulation when compared with the *ACT1* control (**Figure 3B**, *mnr2*). These observations indicate that the increased Alr1 activity observed in Mg-deficient cells of both wild-type and *mnr2* mutant strains were not explained by changes in *ALR1* gene expression.

### Post-translational regulation of Alr1-HA

**Figure 4:**
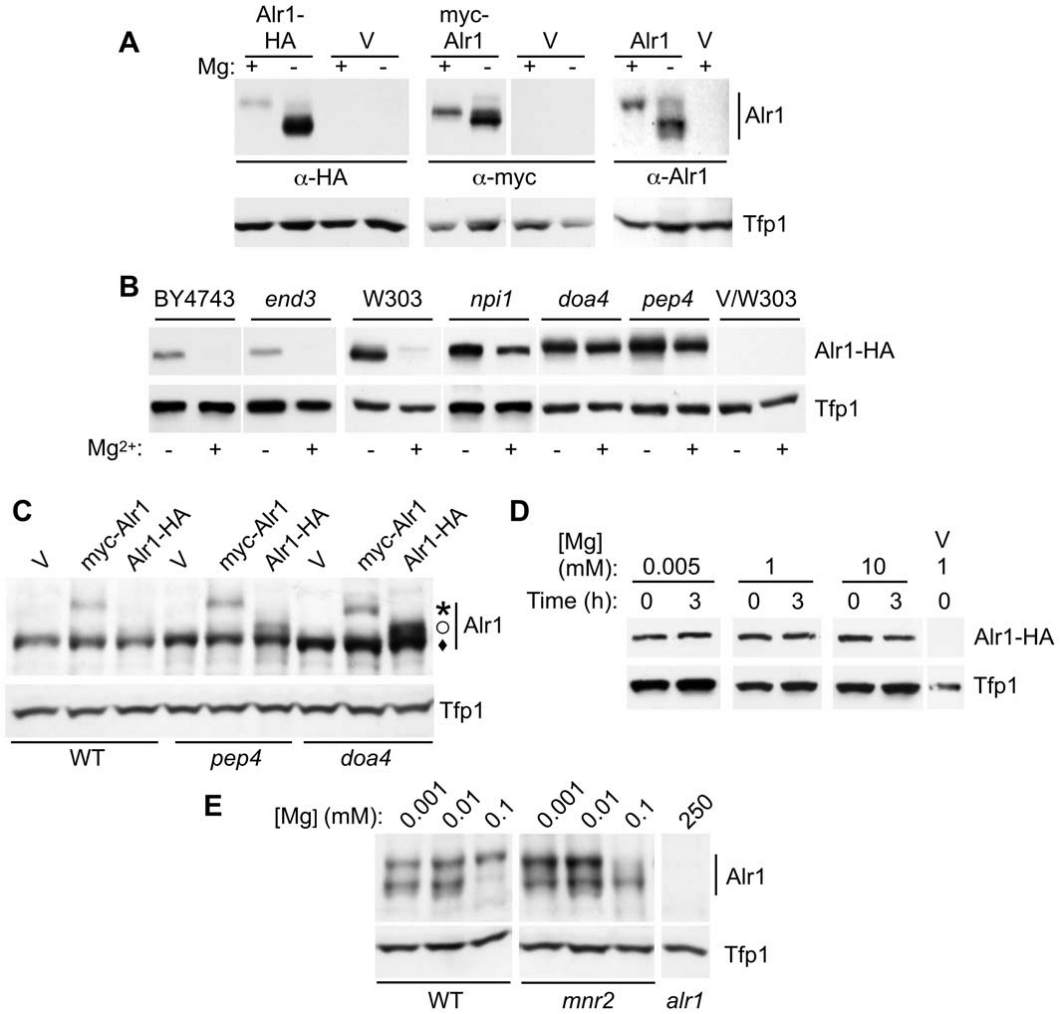
Effect of epitope tagging on Alr1 accumulation. (**A**) DY1457 transformed with YCpALR1-HA (left panel), or YCpmyc-ALR1 (center), and an *alr1* strain (NP10) transformed with YCpALR1 (right), or an empty vector (pFL38, V) were grown in LMM +1 µM (−) or 1 mM Mg (+). Alr1 and Tfp1 (as loading control) were detected by SDS-PAGE and immunoblotting with anti-HA, anti-myc, or anti-Alr1 antibodies. (**B**) Strains of the indicated genotypes transformed with YCpALR1-HA were grown in LMM supplemented with 1 µM (−) or 1 mM (+) Mg, and proteins detected by immunoblotting with anti-HA and anti-Tfp1. Strains used were WT (BY4743 or W303-1B), 32992 (*end3*), SD20 (*doa4*) 27038a (*npi1*), MOB100 (*pep4*), and W303-1B transformed with pFL38 (V/W303). Results for the *NPI1* strain isogenic to 27038a (23344C) were similar to W303-1B (not shown). (**C**) WT (W303-1B), *pep4* (MOB100) and *doa4* (SD20) strains transformed with YCpmyc-ALR1 (myc-Alr1), YCpALR1-HA (Alr1-HA) or pFL38 control (V) were grown in Mg-replete medium (SC). Proteins were detected by immunoblotting with anti-Alr1 and anti-Tfp1. The three versions of Alr1 are indicated (star = myc-Alr1, open circle = Alr1-HA, diamond = untagged Alr1). (**D**) FY1679/YIpALR1-HA cells were grown in LMM containing 5 µM Mg for 12 hours. Cells were then transferred to LMM with 5 µM, 1 mM, or 10 mM Mg, and 100 µg/ml cycloheximide. Aliquots of cells were removed after 0 or 180 minutes, protein extracted, and proteins detected by immunoblotting with anti-HA and anti-Tfp1 antibodies. FY1679 transformed with pFL38 was included to verify antibody specificity (V). (**E**) WT (DY1457), *mnr2* (NP4), and *alr1* (NP10) strains were grown in LMM medium supplemented with the indicated Mg concentration. Proteins were detected by immunoblotting with anti-Alr1 and anti-Tfp1 antibodies.

The above observations suggested that Mg-dependent change in Alr1-HA accumulation (**Figure 2**) was primarily accomplished via post-translational regulation of protein stability [24]. However, before investigating post-translational regulation of Alr1, we verified that the C-terminally HA-tagged version of Alr1 accurately reflected the behavior of the native (untagged) version. To compare the effect of Mg supply on different versions of Alr1, we constructed strains that expressed Alr1-HA, N-terminally myc-tagged Alr1 (myc-Alr1), unmodified Alr1, or no Alr1. All *ALR1* plasmids were single copy vectors that utilized the native *ALR1* promoter. Strains were grown in Mg-deficient and replete conditions, and Alr1 detected by immunoblotting with the appropriate antibodies. Alr1 accumulation was normalized to the Tfp1 loading control to determine the fold changes in Alr1 accumulation discussed below. Consistent with previous experiments (**Figure 2**), we detected an approximate 7-fold difference in the accumulation of the Alr1-HA protein between Mg-replete and deficient cells (**Figure 4A**, left). As previously observed [24], native Alr1 and modified versions migrated as two bands in SDS-PAGE, which may represent differentially phosphorylated forms [25]. Both bands were included when quantifying Alr1 accumulation (clear separation of these bands was dependent on gel polyacrylamide concentration and electrophoresis conditions, meaning discrete bands were not resolved in some experiments, for example **Figure 2A**). In contrast with Alr1-HA, normalized accumulation of myc-Alr1 varied less than 2-fold (**Figure 4A**, center), with a similar minor change observed for the untagged protein (**Figure 4A**, right). These results indicate that the HA tags altered the response of Alr1-HA to Mg supply. However, despite the large effect of Mg supply on Alr1 activity, the accumulation of untagged and N-terminally tagged Alr1 was relatively unresponsive to Mg.

The strong effect of C-terminal epitope tags on Alr1 accumulation was surprising. To further investigate this effect, we examined the consequence of inactivating factors required for ubiquitination, endocytosis, and vacuolar degradation on the accumulation of the three versions of Alr1. A previous study reported that in deficient cells, Alr1-HA stability was substantially reduced by Mg treatment, and that three factors involved in the post-translational regulation of membrane proteins (Pep4, End4 and Rsp5) were required for this process [24]. Rsp5 is an E3 ligase required for the regulated ubiquitination of membrane proteins [48], End4 is required for the endocytosis of post-translationally regulated plasma membrane proteins [49], [50], and Pep4 is required for the maturation of vacuolar proteases essential for the degradation of proteins in the vacuole lumen [51]. We examined the effect of inactivating these and other related factors required for post-translational regulation on the steady state accumulation of the three versions of Alr1 (**Figure 4B**). In wild-type cells of either the BY4743 or W303 genetic background, Alr1-HA accumulated to a substantially higher level in Mg-deficient conditions, as previously observed. In a *pep4* mutant, Alr1-HA accumulated to a much higher level than observed for the Mg-replete wild-type strain. In Mg-deficient *pep4* cells, Alr1-HA accumulated to a similar level to that seen in replete cells. The effect of the *pep4* mutation suggested that in wild-type cells, Alr1-HA was delivered to the vacuole lumen and degraded via a Pep4-dependent mechanism. Since the *pep4* mutation increased Alr1-HA accumulation under both Mg-deficient and replete conditions, Alr1-HA was subject to Pep4-dependent degradation in both conditions, but Mg deficiency appeared to reduce the efficiency of this process.

The Rsp5 E3 ubiquitin (Ub) ligase covalently links Ub to membrane proteins at the plasma membrane and Golgi [52], [53], [54], and was previously reported to be required for the degradation of Alr1-HA in response to Mg repletion [24]. We observed that the *npi1* mutation, which reduces the expression of the essential Rsp5 protein by 90% [52], also substantially increased Alr1-HA accumulation in both Mg-replete and deficient conditions. To further test the effect of inactivating pathways of Ub-dependent protein degradation, we also examined the effect of a *doa4* mutation. Doa4 is a Ub hydrolase that removes Ub from membrane proteins before they are packaged into multi-vesicular bodies (MVB's) at the late endosome [55]. A *doa4* mutant is unable to remove Ub from membrane proteins and sort them into the lumen of the MVB, thereby increasing their stability and accumulation [55]. A *doa4* mutant showed enhanced accumulation of Alr1-HA in both replete and deficient conditions, similar to that observed for *pep4*.

Lastly, we examined the effect of mutating the *END3* gene required for the regulated endocytosis of plasma membrane proteins [56]. End3 is required for the recycling of plasma membrane proteins normally subject to post-translational regulation, such as Gap1 [57] and Zrt1 [35]. Notably, the *end3* mutation did not affect the steady-state level of Alr1-HA accumulation when compared with the isogenic wild-type strain (BY4743), and neither did *end4* or *dim1* mutations, which also block receptor-mediated endocytosis [49], [58] (data not shown). Together, these observations confirmed a role of the Doa4/Pep4-dependent degradation pathway in determining the steady state level of Alr1-HA accumulation in cells. However, they contradict a previous model for Alr1 regulation [24], which argued that Alr1-HA accumulation was regulated in part by Mg-responsive endocytosis.

We then determined the relative degree to which each of the three versions of Alr1 were substrates for the Pep4/Doa4-dependent degradation pathway. Since the *pep4* and *doa4* mutations had the most obvious effect on Alr1-HA accumulation in replete cells, cultures were grown in Mg-replete medium for this comparison. Each form of Alr1 was expressed in wild-type, *doa4* and *pep4* mutant strains, and the accumulation of each was simultaneously detected using an anti-Alr1 antibody. Due to size differences, all three versions of Alr1 could be clearly discriminated (**Figure 4C**), but the results were also verified separately using anti-HA and anti-myc antibodies (data not shown). Both the *doa4* and *pep4* mutations increased the accumulation of the Alr1-HA protein by approximately 6-fold, but each mutation caused less than a 2-fold increase in the accumulation of the myc-Alr1 and untagged proteins. This observation indicated that unlike Alr1-HA, the unmodified or N-terminally tagged proteins were not a major substrate for the Doa4/Pep4-dependent degradation pathway. The effect of these mutations on the latter two versions of Alr1 also indicated that in replete conditions, Alr1 may normally be delivered to the vacuole and degraded at a slow rate. In this respect, Alr1 may behave similarly to tracers of bulk-flow endocytosis (*e.g.* FM4-64), which are slowly transferred from the plasma membrane to the vacuole even in *end3* mutant strains [50]. Our data further indicates that N-terminal tagging does not have the same effect on Alr1 behavior as C-terminal tagging. Although myc-Alr1 accumulated to a lower overall level than the wild-type protein (**Figure 4C**), *doa4* and *pep4* mutations had little effect on its accumulation, and the ratio of the myc-tagged to native protein did not vary in the three strains, indicating that the lower accumulation of Myc-Alr1 may result from an effect of this alteration on variables other than protein stability (for example, transcript stability or translation rate).

Together, these observations indicated that as previously suggested [24], Alr1-HA accumulation is largely determined by the activity of an Rsp5/Doa4-dependent recycling pathway for membrane proteins, and that Mg availability affects the degree to which Alr1-HA is subject to degradation. We suggest that the modification of the Alr1 C-terminus may inhibit correct folding of the protein, or its ability to assemble into a complex with other subunits. If monomeric or misfolded Alr1-HA accumulates in the Golgi, it may be recognized by Rsp5-dependent quality control systems, ubiquitinated, and sorted directly to the vacuole for Pep4-dependent degradation. Similar behavior has been observed for some other defective proteins [38], [40]. In contrast, the accumulation of unmodified Alr1 is much less sensitive to mutations in components of this pathway, suggesting that Ub-mediated degradation does not play a major role in its regulation.

### Effect of Mg on Alr1-HA stability

The lack of effect of the *end3* mutation on Alr1-HA steady state accumulation (**Figure 4B**) suggested that Mg-dependent sorting of Alr1-HA occurred within the cell, and that this process no longer operated on Alr1-HA that had already reached the plasma membrane. This interpretation was however inconsistent with a previous report that the stability of Alr1-HA in deficient cells was Mg-dependent [24]. To clarify this issue, we also determined the effect of Mg supply on Alr1-HA stability using the same protocol (**Figure 4D**). A wild-type strain expressing Alr1-HA was grown in Mg-deficient conditions, then transferred to fresh medium containing 5 µM, 1 mM, or 10 mM Mg, and cycloheximide to block *de novo* protein synthesis. In multiple experiments, we observed no difference in Alr1-HA stability between cells maintained in deficient conditions and those exposed to excess Mg. Our observations directly contradict the previous report, but the reason for this inconsistency is unknown. Alr1-HA insensitivity to Mg was not due to lack of Mg uptake, as robust uptake was directly observed under these conditions (data not shown). The above observations are consistent with the absence of an effect of the *end3* mutation on the steady-state level of Alr1-HA accumulation, as both observations suggest that the stability of Alr1-HA is determined early in the secretory pathway, before the protein reaches the cell surface.

Because the above results indicated that the response of Alr1-HA to Mg supply differed from the untagged protein, we examined the effect of the *mnr2* mutation on the accumulation of the untagged Alr1 protein (**Figure 4E**). Wild-type and *mnr2* cells were grown in deficient (1 and 10 µM) and replete (100 µM) Mg concentrations and Alr1 detected via immunoblotting. In contrast to Alr1-HA (**Figure 2**), cells expressing native Alr1 and supplied with 1 µM Mg accumulated less than 2-fold more Alr1 protein than cells supplied with 100 µM Mg (**Figure 4E**), although they displayed approximately 9-fold higher Alr1 activity (**Figure 1C**). In addition, while the *mnr2* mutant supplied with 100 µM Mg had approximately 9-fold higher Alr1 activity than wild-type cells (**Figure 1C**), the mutation had little effect on the abundance of the Alr1 protein under the same conditions (**Figure 4E**). These observations indicate that a change in Alr1 accumulation alone does not explain the substantial change in Alr1 activity elicited by loss of Mnr2 function.

### Alr1 location and Mg supply

**Figure 5:**
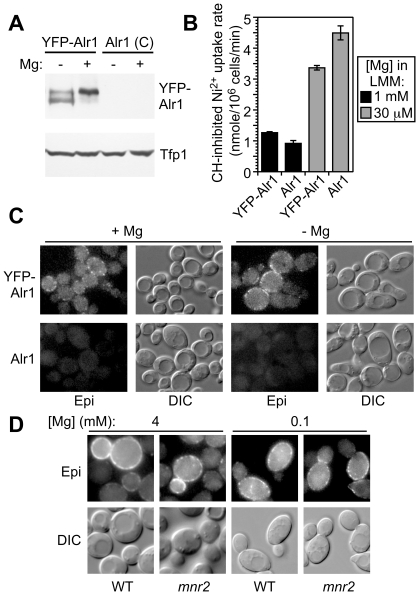
Effect of Mg supply on Alr1 location. (**A**) *alr1* strains (NP4) transformed with YCpCit-ALR1 (YFP-Alr1) or YCpALR1 (Alr1) were grown for 16 h in LMM supplemented with 1 µM (−) or 1 mM (+) Mg prior to protein extraction. YFP-Alr1 and Tfp1 proteins were detected by immunoblotting with anti-GFP or anti-Tfp1 antibodies respectively. (**B**) Rate of CH-inhibited Ni^2+^ uptake by cells expressing native and YFP-tagged Alr1. Strains described in (A) were grown to log phase in SC medium, washed to remove Mg, and incubated for 6 h in LMM with the indicated Mg concentration. The rate of CH-inhibited Ni^2+^ uptake was then determined as described in the legend to **Figure 1**. Values are means of four replicates +/−1 SEM. (**C**) Strains described in (A) were grown to exponential phase in SC, then transferred to LMM containing 5 µM (−) or 5 mM (+) Mg for 6 hours, and live cells examined by epifluorescence microscopy. YFP fluorescence (Epi) and corresponding differential interference contrast (DIC) images are shown. (**D**) Diploid WT (BY4743) and *mnr2* (34913) strains transformed with YCpCit-ALR1 were grown to log phase in SC medium (2 mM Mg), or LMM+100 µM Mg and examined with epifluorescence microscopy (Epi), or DIC as indicated.

Since the untagged and N-terminally tagged versions of Alr1 did not display behavior consistent with substantial post-translational regulation of stability or accumulation, we considered the possibility that endocytosis contributed to the regulation of Alr1 activity, but without affecting its steady-state accumulation. For example, a process of Mg-dependent trafficking could operate to reduce Alr1 level at the cell surface in Mg-replete conditions, but transfer it back to the surface in deficient cells. To test this model, we directly examined the effect of Mg supply on subcellular Alr1 location, using a functional N-terminal fusion of Alr1 to YFP. Under steady state conditions, YFP-tagged Alr1 expressed from the *ALR1* promoter accumulated to a similar level in Mg-deficient and replete conditions (**Figure 5A**), and Ni^2+^ uptake experiments confirmed that the activity of the YFP-tagged protein was regulated by Mg supply (**Figure 5B**). When expressed from the *ALR1* promoter, YFP-Alr1 was detected as a faint punctate signal on the plasma membrane, and its location was not noticeably altered by Mg status (**Figure 5C**). Thus, we found no evidence that Alr1 activity was regulated by altering YFP-Alr1 protein distribution. We also examined the effect of the *mnr2* mutation on YFP-Alr1 location. Although the *mnr2* mutation increased Alr1 activity approximately 9-fold in cells supplied with 100 µM Mg (**Figure 1C**), the mutation had no effect on the location of YFP-Alr1 in cells supplied with either 100 µM or 4 mM Mg (**Figure 5D**), indicating that a change in YFP-Alr1 distribution did not explain the increased activity observed in *mnr2*.

## Discussion

The CorA family of Mg channels is widespread in biology, and its members play an important role in the regulation of cytosolic Mg concentration. Although many CorA proteins have been shown to be constitutively expressed [59], a previous study suggested that Alr1 expression was Mg-regulated [24]. In this report, we used both inhibitors of the Alr systems and Alr mutant strains to establish for the first time that Ni^2+^ uptake by *S. cerevisiae* is primarily dependent on Alr1, and that this activity is substantially increased by Mg deficiency. We also report that Alr1 activity was increased in a mutant (*mnr2*) lacking access to intracellular Mg stores, consistent with regulation by intracellular Mg concentration. To understand how Alr1 activity is regulated, we examined the effect of Mg supply on Alr1 expression and accumulation. In contrast to a previous study, we found no evidence for Mg-regulated expression of the *ALR1* gene. The accumulation of an epitope-tagged version of Alr1 (Alr1-HA) was found to be dependent on Mg supply and on proteins required for ubiquitin-dependent protein sorting and degradation, but these factors had little influence on the accumulation of the native protein. We also observed that although the *mnr2* mutation increased Alr1 activity, this change was not explained by altered accumulation of the Alr1 protein or its redistribution. Some implications of these findings are discussed below.

### Effect of Mg on ALR1 gene expression

Using Northern analysis, we observed that *ALR1* gene expression was insensitive to Mg supply (**Figure 3**). We also used an independent method (an *ALR1* promoter-*lacZ* fusion construct), to confirm the insensitivity of the *ALR1* promoter to Mg availability. Thus, we are confident that these results accurately reflect the effect of Mg on *ALR1* gene expression. We are unsure as to why we could not repeat earlier observations of an effect of Mg on *ALR1* transcript levels, but we note that the small effect of Mg supply on native Alr1 protein accumulation is consistent with a lack of *ALR1* transcript regulation. A previous report [25] also indicated that *ALR2* gene expression was upregulated by Mg deficiency. Although we did not examine the effect of Mg on *ALR2* mRNA abundance or protein accumulation, there are several reasons why it is unlikely that *ALR2* upregulation alone could explain the large increase in Alr system activity under Mg-deficient conditions. First, several studies have shown that both *ALR2* gene expression and protein accumulation is very low [25], [26], [27]. Indeed, our attempts to detect *ALR2* mRNA by Northern analysis were unsuccessful (data not shown). This low expression may be explained by a recent study showing the *ALR2* mRNA is targeted for nonsense-mediated decay [27]. The sequence of Alr2 also varies from Alr1 at a key residue, reducing its activity [25]. Consistent with these observations, the inactivation of *ALR1* alone is sufficient to induce a strong Mg-dependent growth phenotype, even under deficient conditions that might be predicted to induce *ALR2* expression [24], [26], [29]. In addition, the inactivation of *ALR1* alone resulted in a substantial decrease in cellular Mg content even under Mg-replete conditions [29]. Together, these observations indicate that Alr2 activity alone is not sufficient to maintain Mg homeostasis.

### Effect of Mg on Alr1 accumulation

**Figure 6:**
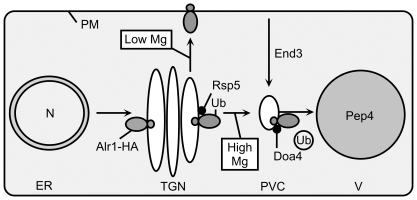
Proposed model for Mg-dependent trafficking of Alr1-HA. After synthesis, fate of the Alr1-HA protein is dependent on Mg supply. In Mg-deficient cells, more Alr1-HA escapes an Rsp5-dependent quality control mechanism and reaches the plasma membrane, where it is not subject to Mg-stimulated endocytosis. In replete conditions, Alr1-HA is recognized by Rsp5, ubiquitinated, and sorted to the PVC, where it is intercepted by Doa4 and sorted to the vacuole lumen for eventual Pep4-dependent degradation.

Since our data did not support regulation of *ALR1* gene expression by Mg, we examined the effect of Mg supply on Alr1 protein accumulation (**Figure 4**). Although we could reproduce the effect of Mg supply on the accumulation of an epitope-tagged Alr1-HA protein (**Figure 2**), our data indicate that this effect is an artifact of adding C-terminal epitope tags to Alr1, because neither the untagged, nor N-terminally tagged versions of Alr1 were similarly regulated. Based on these observations, we suggest a model to explain the effect of Mg on the accumulation of the tagged Alr1-HA protein (**Figure 6**). Our data indicate that this modification decreased Alr1 abundance by increasing its susceptibility to systems that degrade misfolded proteins. We suggest that while some Alr1-HA reaches the plasma membrane and is functional, a large fraction is sorted directly from the Golgi to the vacuole, where it is degraded. This process is accelerated under Mg-replete conditions, leading to reduced accumulation of Alr1-HA. The involvement of Rsp5 and Doa4 in determining the level of Alr1-HA accumulation suggests that ubiquitination of Alr1-HA by Rsp5 early in the secretory pathway triggers its sorting to the vacuole. The instability of newly synthesized Alr1-HA may result from the HA tag inhibiting the assembly of subunits into a functional complex, leading to the accumulation of unstable monomeric forms. A previous study reported that the deletion of the last 36 amino acids of the Alr1 C-terminal domain reduced the ability of the protein to homo-oligomerize [25] suggesting that modification of this region with HA tags might also inhibit assembly. Since the Alr1-HA protein that accumulated in Mg-deficient cells was not destabilized by subsequent exposure to Mg-replete conditions (**Figure 4D**), Alr1-HA that escapes Rsp5-dependent quality control mechanisms within the secretory pathway may take on a stable conformation that is resistant to endocytosis and degradation. In support of this model, we note that Mg repletion did not reduce the stability of the pool of preexisting Alr1-HA in Mg-deficient cells (**Figure 4D**), and the *end3* mutation had no effect on steady-state Alr1-HA accumulation (**Figure 4B**). These observations suggest that in replete conditions, Alr1-HA does not reach the plasma membrane en route to the vacuole, and its degradation is thus not dependent on Mg-regulated endocytosis.

Unlike Alr1-HA, our experiments indicated that native and N-terminally tagged versions of Alr1 are not major targets for Mg-dependent ubiquitination, trafficking, and degradation. Additional evidence for this view came from examination of YFP-Alr1 location in mutant strains defective for membrane protein turnover (**Figure S1**). The response of YPF-tagged Alr1 to Mg supply was similar to both the native and N-terminally myc-tagged proteins (**Figure 4**, **Figure 5**), indicating that this protein could be used to examine Alr1 trafficking. In Mg-replete conditions, YFP-Alr1 was predominantly located at the cell surface of wild-type cells (**Figure S1A**). The *npi1* mutation did not increase the level of YFP-Alr1 localized to the plasma membrane, as might be expected if Rsp5-dependent ubiquitination regulated Alr1 stability [24]. In addition, YFP-Alr1 did not accumulate in the vacuole of *pep4* mutant cells lacking vacuolar proteases. We also examined the effect of a *vps27* mutation, which blocks the transport of ubiquitinated membrane proteins from the prevacuolar compartment (PVC) to the vacuole [60]. Substantial transport of YFP-Alr1 from the plasma membrane to the vacuole would cause YFP-Alr1 to accumulate in the PVC of a *vps27* mutant, which was not observed (**Figure S1A**). Together, these findings provide further evidence that post-translational regulation does not influence the fate of Alr1.

If the instability of the Alr1-HA protein is an artifact of its tagging, why then is Alr1-HA accumulation responsive to Mg supply? If key steps in protein quality control mechanisms were Mg dependent, these processes might become less efficient in Mg-deficient cells. Consistent with this idea, many of the basic processes required for protein trafficking and ubiquitination are ATP-dependent (*e.g.*, the covalent addition of ubiquitin to E1 ligase enzymes) [61], and may thus depend on access to adequate Mg as a cofactor. Another possible explanation is that the conformation of the Alr1-HA protein itself may be Mg-dependent, which could directly affect its stability. CorA from *Thermatoga maritima* undergoes large-scale conformational changes upon binding Mg, which play a role in regulating its activity [62], [63]. Many of the Mg-binding residues required for these conformation changes are conserved in the Alr1 protein (data not shown), suggesting that Mg availability may have a similar effect on Alr1 conformation. If so, it is possible that when Alr1-HA binds Mg it abnormally exposes certain residues, thereby increasing its visibility to protein quality control mechanisms. Alternatively, the protein may take on an aberrant conformation which inhibits its ability to assemble into a functional complex, with similar consequences for stability.

Precedent for this model (**Figure 6**) comes from studies of plasma membrane proteins such as the Smf1 divalent cation transporter [64], [65], [66]. In Mn^2+^-deficient conditions, Smf1 accumulates in the plasma membrane [67], but in replete conditions, this protein moves directly from the Golgi to the vacuole and is degraded. This trafficking pathway requires Smf1 ubiquitination, but this modification happens early in the secretory pathway rather than at the plasma membrane [68]. Like Alr1-HA, delivery of newly synthesized Smf1 to the vacuole did not require endocytosis, as the *end4* mutation had no effect on Smf1 stability in replete conditions [67]. The Gap1 amino acid permease is regulated via a similar mechanism [53], [69], [70], [71]. The pathway responsible for directing both Smf1 and Gap1 to the endosome shares components with a general quality control mechanism that eliminates misfolded membrane proteins from the secretory pathway. The Pma1-7 protein, a temperature-sensitive mutant form of the plasma membrane ATPase, also reaches the vacuole via this pathway in non-permissive conditions [38], [72]. Thus, the existence of this sorting pathway and its involvement in both the regulation and recycling of damaged proteins is well established.

### Regulation of the Alr systems

Given that regulated abundance does not explain the effect of Mg on Alr1 activity, what other processes might explain this regulation? Structural studies of a bacterial CorA protein revealed that its conformation was altered by the presence of Mg ions bound to intracellular sites [62], [63], [73], [74]. The N-terminal cytosolic domain of the CorA protein includes two cation-binding sites located at the boundaries of each adjacent subunit. The presence of Mg was associated with rearrangement into a more compact protease-resistant conformation, and the apparent closure of the ion transport pore. This observation suggests that CorA proteins form Mg-gated pores in the membrane, with the cytosolic domain acting as a Mg-sensor. This model is supported by electrophysiological studies showing Mg-dependent activity of both CorA and Mrs2 channels [17], [62]. We suggest that activity of the Alr1 protein may also be directly regulated by binding cytosolic Mg. The effect of the *mnr2* mutation on Alr1 activity is consistent with this model, since this mutation is likely to lower cytosolic Mg availability.

It is also possible that other regulatory processes affect Alr1 activity. During this work we consistently observed an effect of Mg supply on the speed of Alr1 migration in SDS-PAGE (**Figure 4A** **,** **Figure 5A**). A higher mobility form of Alr1 predominated in deficient cells, while a lower mobility form was seen in cells supplied with >10 µM Mg. This apparent modification of Alr1 in response to Mg supply has been observed in several studies [24], [25], [41], and was seen with all versions of Alr1 used here. On the basis of the effect of treatment with protein phosphatase on Alr1 gel mobility, one report indicated that the lower mobility form may be phosphorylated [25]. Consistent with this explanation, proteomic studies identified several phosphorylated residues in Alr1 [75]. If phosphorylation of the Alr1 protein is Mg-dependent, this modification might play a role in adaptation to Mg-deficient conditions, perhaps by directly regulating Alr1 activity. In support of this model, we note that cells of the *mnr2* mutant strain supplied with 100 µM Mg predominantly accumulated the higher mobility form of Alr1, while the wild-type predominantly displayed the lower mobility form (**Figure 4E**). The predominant form of Alr1 thus correlated well with Alr1 activity (**Figure 1C**). The nature and effect of this modification represents an interesting subject for future studies of Alr1 regulation.

In summary, we have identified a mechanism of regulation of the Alr1 protein activity by intracellular Mg supply, which may contribute to Mg homeostasis in yeast. However, our work does not support a major role for the regulation of *ALR1* gene expression or protein stability in this process. In addition to expanding our understanding of the function of the Alr1 and Mnr2 proteins in Mg homeostasis, this report provides a cautionary tale about the use of functional tags to modify proteins. Both N- and C-terminally tagged versions of Alr1 were functional, as determined by complementation assays (**Figure S1B**), but displayed very different responses to Mg supply. Such growth assays are often the only evidence presented to argue that epitope tags do not modify the function or behavior of a protein. However, it is clear from this work that epitope tagging can have subtle, and even misleading effects on protein behavior.

## Materials and Methods

### Yeast strains, growth media and standard methods

All yeast strains used in this work are listed in **Table 1**. Yeast strains were routinely propagated as described previously [29]. For culture of strains in Mg-deficient conditions, a low magnesium synthetic medium (LMM) was prepared [29]. Yeast transformation was performed using standard methods [76]. β-galactosidase activity was measured by the method of Guarente [77]. Cells were harvested during exponential growth and activity was calculated as follows: (*A*_415_×1000)/(min×ml of culture used×culture *A*_595_). Cell number per milliliter of yeast suspensions was determined by measuring the optical density at 595 nm (*A*_595_) and comparing with a standard curve of *A*_595_ vs. viable cells.

**Table 1:** Yeast strains.

Strain	Full genotype	Source/reference
DY1457	*MAT* **a** *ade6 can1-100^oc^ his3-11,15 leu2-3,112 trp1-1 ura3-52*	D. Eide [85]
NP4	*mnr2::KanMX4* in DY1457	[29]
NP10	*MAT* **a** *ade2 can1-100^oc^ his3-11,15 leu2-3,112 trp1-1 ura3-52 alr1::HIS3*	[29]
NP27	*MAT* **a** *can1-100^oc^ his3-11,15 leu2-3,112 trp1-1 ura3-52 alr2::TRP1*	[29]
NP14	*MAT* **a** *ade6 can1-100^oc^ his3-11,15 leu2-3,112 trp1-1 ura3-52 alr1::HIS3 alr2::TRP1*	[29]
NP20	*MAT* **a** *ade2 can1-100^oc^ his3-11,15 leu2-3,112 trp1-1 ura3-52 alr1::HIS3 alr2::TRP1 mnr2::KanMX4*	[29]
W303-1B	*MATα ade2-1 ura3-1 his3-11 leu2-3,112 trp1-1 can1-100*	R. Haguenauer-Tsapis [86]
MOB100	*MATα ade2-1 ura3-1 his3-11 leu2-3,112 trp1-1 can1-100 pep4::KanMX4*	R. Haguenauer-Tsapis [86]
SD20	*MATα ade2-1 ura3-1 his3-11 leu2-3,112 trp1-1 can1-100 doa4::HIS3*	R. Haguenauer-Tsapis [86]
BY4743	*MAT* **a** */MATα his3Δ1/his3Δ1 leu2Δ0/leu2Δ0 met15Δ0/MET15 lys2Δ0/LYS2 ura3Δ0/ura3Δ0*	EUROSCARF [87]
34913	*mnr2::KanMX4/mnr2::KanMX4* in BY4743	EUROSCARF [87]
35381	*vps27::KanMX4/vps27::KanMX4* in BY4743	EUROSCARF [87]
32992	*end3::KanMX4/end3::KanMX4* in BY4743	EUROSCARF [87]
23344c	*MATα ura3*	B. Andre [88]
27038a	*MAT* **a** *npi1 ura3*	B. Andre [88]
FY1679	*MAT* **a** *ura3-52*	A. Graschopf [24]
SF838-9D	*MAT* **a** *pep4-3 leu2-3,112 ura3-52 his4-519 gal2*	R. Piper [89]

### Plasmid construction

All plasmids were constructed via gap repair in yeast [78], and complete sequences are available on request. The plasmids pFL38 [79], YIpALR1-HA [24], YEp353 [80], and YCpALR1 [26] were described previously. YEpALR1-lacZ was constructed by amplifying the 5′ intergenic region of *ALR1* including the start codon from genomic DNA. This fragment was fused 5′ of the *lacZ* ORF via gap repair of YEp353. YCpALR1-HA was constructed by transferring the *ALR1* promoter and complete coding sequence (including the HA tags) from the YIpALR1-HA plasmid to pFL38 via gap repair. The YEpGmycALR1 plasmid was constructed from YEpGmyc-Mnr2 [29], by replacement of the *MNR2* ORF with *ALR1* via gap repair. This construct contains six repeats of the c-myc epitope sequence fused to the start of the *ALR1* ORF. To express myc-tagged Alr1 from the *ALR1* promoter, the six myc tags and the 5′ end of the *ALR1* ORF were amplified from YEpGmycALR1 and integrated between the *ALR1* promoter and coding sequence in the YCpALR1-HA plasmid, generating YCpmyc-ALR1-HA. The HA tags were then removed by replacement with a PCR fragment of the *ALR1* C-terminus, generating YCpmyc-ALR1. To construct YEpGCit-Alr1, the coding sequence of the YFP gene (yEmCitrine variant) was amplified from the pKT211 vector [81] and used to replace the N-terminal myc tags in YEpGmycALR1 via gap repair. The resulting plasmid expressed YFP-tagged Alr1 from a galactose-regulated promoter. To construct YCpCit-ALR1, the 5′ region of the YEpGCit-Alr1 plasmid including the YFP ORF and an N-terminal portion of *ALR1* was amplified and inserted at the N-terminal end of the Alr1 ORF in the YCp-myc-Alr1 plasmid. Gap repair reconstituted a plasmid in which six myc tags and the YFP gene are inserted between the *ALR1* promoter and start of the *ALR1* CDS. The function of all modified Alr1 proteins was verified by complementation of the growth defect of an *alr1 alr2* mutant strain (**Figure S1**).

### Northern blotting and hybridization

RNA was extracted from yeast using standard methods [82]. mRNA was purified from total RNA using a GenElute miniprep kit (Sigma). Formaldehyde agarose gel electrophoresis, Northern blotting, and hybridization procedures were performed using standard methods [83]. Typically, twenty µg of mRNA was loaded per lane and fractionated on a 1% agarose formaldehyde gel. RNA was blotted to Hybond N membrane prior to hybridization with ^32^P-labelled PCR products. Autoradiographs were visualized using a phosphorimager.

### SDS-PAGE, immunoblotting and microscopy

SDS-PAGE and immunoblotting experiments were performed as previously described [29]. Antibodies used in these studies were rabbit anti-myc (ab9106, Abcam), mouse monoclonal anti-HA (ab 9110, Abcam), mouse anti-Tfp1 (8B1, Molecular Probes), rabbit anti-GFP (ab290, Abcam), HRP-conjugated goat anti-mouse (32230, Pierce), and HRP-conjugated goat anti-rabbit (32260, Pierce). To generate the anti-Alr1 antibody, a GST-fusion to the C-terminal 230 amino acids of Alr1 was expressed in *E. coli*, purified, and used to immunize rabbits (R. Gardner, unpublished results). For visualization of YFP fluorescence, live cells of log-phase cultures were applied to poly-L-lysine coated slides and examined with a Zeiss Axioscope fluorescence microscope equipped with Zeiss FL Filter Set 46 HE to detect YFP emission. Images were captured with a Retiga EXi cooled charge-coupled device camera (QImaging) using IP Labs 4.0 software (BD Biosciences). Images were edited to filter noise and adjust contrast using Photoshop CS (Adobe Systems). Immunoblot band density was determined from TIFF images using ImageJ 1.36b software.

### Elemental analysis

ICP-MS analysis was performed essentially as previously described [84], except that after collection, yeast cells were washed with 1 mM EDTA and water, then resuspended in uptake buffer (10 mM Tris-succinate pH 4, 2% glucose). Uptake assays were initiated either by the addition of cells to buffer containing metal ions (*e.g.* Ni^2+^), or by the addition of metal ions to cell suspensions. Assays were terminated by adding EDTA to 10 mM and chilling the cells on ice. Cells were collected on nitrocellulose filters, then washed twice with wash buffer (10 mM Tris-succinate pH 4, 1 mM EDTA), twice with ddH_2_O, and processed for ICP-MS.

## Supporting Information

Figure S1 **Subcellular location and function of epitope-tagged Alr1.** (**A**) Effect of protein trafficking mutations on YFP-Alr1 location. WT (W303-1B), *pep4* (SF838-9D), *vps27* (35381), and *npi1* (27038a) yeast strains were transformed with a *GAL1* promoter-driven YFP-Alr1 construct (YEpGCit-Alr1) and strains grown to log phase in SC-U medium (2% glucose) before examination with epifluorescence microscopy (Epi) or DIC as indicated. (**B**) Complementation of an *alr1 alr2* mutant by modified Alr1 proteins. NP14 strains (*alr1 alr2*) transformed with the indicated plasmids were grown to saturation in high Mg medium (SC+250 mM Mg), and used to inoculate aliquots of LMM+30 µM Mg to give an initial *A*_595_ of 0.01. Cultures were grown for 16 h before recording final *A*_595_ values (values are means of three replicate cultures, error bars indicate +/−1 SEM).
(PDF)
