# Supplementary figures and images for: Regulation of Alr1 Mg Transporter Activity by Intracellular Magnesium
Source: PLoS One. 2011 Jun 28;6(6):e20896. doi: 10.1371/journal.pone.0020896 (PMC3125163; doi:10.1371/journal.pone.0020896)

Figure S1

Lim et al 2011

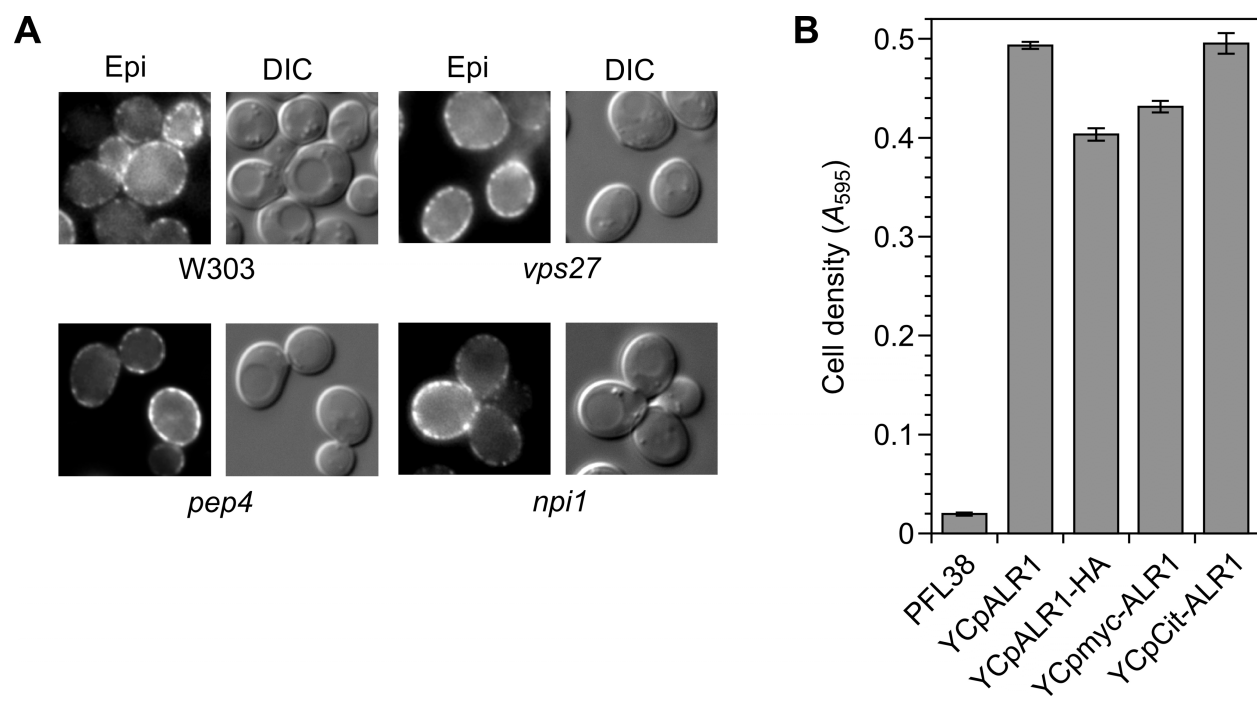

Supplement: Figure S1 — Subcellular location and function of epitope-tagged Alr1. (A) Effect of protein trafficking mutations on YFP-Alr1 location. WT (W303-1B), pep4 (SF838-9D), vps27 (35381), and npi1 (27038a) yeast strains were transformed with a GAL1 promoter-driven YFP-Alr1 construct (YEpGCit-Alr1) and strains grown to log phase in SC-U medium (2% glucose) before examination with epifluorescence microscopy (Epi) or DIC as indicated. (B) Complementation of an alr1 alr2 mutant by modified Alr1 proteins. NP14 strains (alr1 alr2) transformed with the indicated plasmids were grown to saturation in high Mg medium (SC+250 mM Mg), and used to inoculate aliquots of LMM+30 µM Mg to give an initial A 595 of 0.01. Cultures were grown for 16 h before recording final A 595 values (values are means of three replicate cultures, error bars indicate +/−1 SEM). (PDF) [file pone.0020896.s001.pdf]
